# The challenges of maintaining genetic privacy

**DOI:** 10.7554/eLife.54467

**Published:** 2020-01-07

**Authors:** Shai Carmi

**Affiliations:** Braun School of Public Health and Community MedicineThe Hebrew University of JerusalemJerusalemIsrael

**Keywords:** identity by state, identity by descent, genetic genealogy, genetic privacy, Human

## Abstract

Two studies suggest that a determined adversary may be able to obtain genetic information without permission from some genealogy databases.

**Related research article** Edge MD, Coop G. 2020. Attacks on genetic privacy via uploads to genealogical databases. *eLife* **9**:e51810. doi: 10.7554/eLife.51810

The direct-to-consumer genetic testing industry has grown rapidly in the past few years, to the extent that the companies offering such tests now hold a large proportion of all the human genetic data ever generated (Regalado, 2019). A common reason why someone might undergo genetic testing is to discover relatives, either within the database of the company that performed the test, or via one of a number of third-party services that allow users to upload genomes generated by other labs. Two new studies demonstrate that it may be possible for a user to obtain genomic data without permission from some databases (Edge and Coop, 2020; Ney et al., 2020).

In general, when a user uploads their genome to a third-party service, the service searches its database for genomes that have segments that are identical or nearly identical to segments of the user's genome. The number of such identical-by-state (IBS) segments, and the length of these segments, both increase with the closeness of the relationship between the user and the person (or persons) in the database. The minimum length of a segment is typically around a few millions of base pairs.

To see how a user could access data they should not be able to access, suppose that Alice uploads her genome and finds that she is related to Bob. If the testing service gives Alice details about the IBS segments she shares with Bob (such as the location of these segments in the genome), then Alice will have obtained a certain amount of genomic information about Bob. Now, two independent groups – Michael Edge and Graham Coop of the University of California, Davis writing in eLife (Edge and Coop, 2020), and Peter Ney, Luis Ceze, and Tadayoshi Kohno of the University of Washington in work to be presented at the NDSS symposium in San Diego in February (Ney et al., 2020) – report how services that give users certain details about IBS segments could be subject to attacks that allow an 'adversary' to obtain potentially significant amounts of genomic information that they should not have permission to access (Edge and Coop, 2020; Ney et al., 2020).

The key insight is that an adversary does not have to upload their own genome, and that they can instead upload multiple genomes, including genomes that are in the public domain. This approach is called 'IBS tiling'. For each IBS segment that is reported, the adversary gains a small amount of genetic information about a 'target' genome in the database. However, by uploading a large number of genomes, it is possible to obtain large amounts of genetic information (Figure 1A). Using simulations, Edge and Coop showed that with about 900 public genomes from the 1000 Genomes Project, IBS tiling is expected to reveal about 60% of the genome of a European target. A related approach developed by Edge and Coop, named 'IBS probing', allows the adversary to learn if the target's genome contains a specific disease allele (Larkin, 2017; Figure 1B).

**Figure 1.**
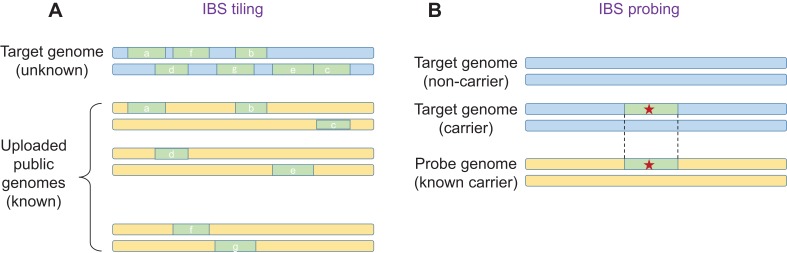
IBS tiling and IBS probing. (**A**) In IBS tiling a user (called the 'adversary') uploads multiple public genomes (shown in yellow) to a DNA matching service in order to determine the sequence of a target genome (pale blue) that is already present in the service's database. In the figure, uploading the first genome yields three IBS segments (a,b,c; pale green), uploading the second genome yields two (d,e), and uploading the third genome also yields two (f,g). IBS tiling only works if the matching service reports matching IBS segments and their locations between the public genomes and the target genome (see text). The amount of information obtained by the adversary increases with the number of public genomes uploaded to the service. (**B**) In IBS probing, the adversary uploads a 'probe' genome that belongs to a person who is known to carry an important mutation (such as a mutation that causes a disease; red star). If the target genome contains the same mutation, the DNA matching service will (under certain conditions) report a matching IBS segment, and the adversary will know that the target also has this mutation in their genome. In general, IBS probing is expected to work for mutations that are relatively young (that is, less than about 500–1000 years old).

The risk of IBS tiling and IBS probing is limited in services that only report IBS segments to users who are closely related. Thus, as genomes from public databases will only rarely be close relatives of the target, this will limit the effective number of genomes available for tiling. However, IBS tiling could yield significant amounts of information on targets from founder populations in which the rate of genomic sharing is high, such as Ashkenazi Jews or Finns (Carmi et al., 2014; Martin et al., 2018). Direct-to-consumer genetic testing companies and third-party services could eliminate this risk by not showing users where IBS segments are located within the genome.

The most popular third-party service, GEDmatch, has over a million users, and was recently acquired by the forensics genomics company Verogen (Husbands, 2019). GEDmatch puts very few restrictions on users and is vulnerable to IBS tiling. GEDmatch is routinely used by police forces to investigate crime (Erlich et al., 2018; Kennett, 2019), though (as of recently) they can only search the genomes of users who have opted in to give law-enforcement agencies access to their genetic information.

When comparing genomes, GEDmatch uses a simple algorithm, reporting a region of the genome as an IBS segment so long as the user and the target do not have conflicting homozygous genotypes: that is, if the user genome is, say, AA at a given site, GEDmatch will return an IBS segment if the target is AA or AB at that site, but not if the target is BB (subject to the segments being longer than a certain minimum length, as described above). GEDmatch also provides users with an image, indicating, for each site in the genome, whether the genotypes of the user and the target fully match, partly match, or do not match.

Ney et al. recently demonstrated that it is possible to extract nearly the entire genome of an individual from GEDmatch by uploading an artificial nearly-all-heterozygote genome and examining the resulting IBS segments (which was also shown by Edge and Coop), or by uploading an all-homozygote genome and examining the resulting images. However, these techniques depend crucially on the specifics of the genome comparison methods used by GEDmatch, and could become obsolete if these methods change, or if users are prohibited from uploading artificial or manipulated genomes.

The use of digital signatures could also prevent adversaries from uploading genomes they have downloaded from public resources or have generated computationally (Erlich et al., 2018). This would involve direct-to-consumer genetic testing labs digitally signing their genome files before users can download them, and third-party services only returning information about IBS segments to a user if the genome uploaded by the user has a digital signature from an approved lab.

The practical consequences of an adversary getting access to your genetic information are debatable. For example, some researchers question the potential usefulness of methods that predict the risk of disease based on polygenic scores (Wald and Old, 2019), especially for non-European populations (Martin et al., 2019). However, others argue for a clinical utility of polygenic risk scores (Lambert et al., 2019). Likewise, there are contrasting views on the usefulness of information about mutations in protein-coding regions. For example, some argue that most coding mutations carried by an individual are difficult to interpret, even by physicians (Hoffman-Andrews, 2017). However, databases such as ClinVar allow users to interpret the pathogenicity of many mutations, and some mutations can be strong risk factors for diseases such as Alzheimer's or breast cancer, which may affect insurance decisions.

However, one needs to remember that DNA is immutable, and thus, any loss of privacy cannot be reversed. Moreover, any loss of privacy can go beyond the individual and extend to their relatives. Further, if an entire large US-based database was compromised, an adversary would be able to identify most US individuals, even those not in the database (Erlich et al., 2018). Therefore, I urge all stakeholders to pay attention to the work of these two groups and attempt to keep genetic information secure.
